# The Diagnostic Value of sTWEAK in Acute Ischemic Stroke

**DOI:** 10.4274/balkanmedj.galenos.2020.2020.2.45

**Published:** 2020-10-23

**Authors:** Ertan Comertpay, Sevilay Vural, Oğuz Eroğlu, Nermin Dindar Badem, Yasemin Karadeniz Bilgili, Figen Coşkun

**Affiliations:** 1 Department of Emergency Medicine, Kırıkkale University School of Medicine, Kırıkkale, Turkey; 2 Department of Emergency Medicine, Bozok University School of Medicine, Yozgat, Turkey; 3 Department of Clinical Biochemistry, Kırıkkale University School of Medicine, Kırıkkale, Turkey; 4 Department of Radiology Kırıkkale University School of Medicine, Kırıkkale, Turkey

**Keywords:** Ischemic area volume, ischemic stroke, magnetic resonance imaging, sTWEAK

## Abstract

**Background:**

Considering the critical role of early diagnosis and management of acute ischemic stroke, biomarkers that can reliable assist in the diagnosis are still needed. These biomarkers should rapidly analyze, have high specificity for brain damage, and be available in the emergency settings for early diagnosis and exclusion of other conditions that mimic acute ischemic stroke. Soluble tumor necrosis factor-like weak inducer of apoptosis, a protein involved in the regulation of several biological functions, could be a potential acute ischemic stroke biomarker.

**Aims:**

To investigate the diagnostic value of soluble tumor necrosis factor-like weak inducer of apoptosis in patients with acute ischemic stroke and examine the relationship between ischemic area volume determined at diffusion-weighted magnetic resonance imaging and soluble tumor necrosis factor-like weak inducer of apoptosis.

**Study Design:**

A prospective, case-control study.

**Methods:**

This case-control prospective study included 36 patients with acute ischemic stroke and 36 healthy volunteers. Information on age, sex, presence of chronic disease, neurological examination findings, times of presentation to the emergency department after acute ischemic stroke, soluble tumor necrosis factor-like weak inducer of apoptosis levels, ischemic area volumes at diffusion-weighted magnetic resonance imaging, and 6-month mortality rates after stroke were recorded. The results were analyzed on SPSS 22.0 software (SPSS Inc., Chicago, IL, USA), and p<0.05 was considered statistically significant.

**Results:**

A soluble tumor necrosis factor-like weak inducer of apoptosis cut-off value of 995.5 pg/mL exhibited a sensitivity of 80.5% and a positive predictive value of 82.5% with an area under the curve of 0.84 (95% confidence interval: 0.74-0.94; p<0.001). The mean soluble tumor necrosis factor-like weak inducer of apoptosis levels in the acute ischemic stroke group (1968.08±1441.99 μg/L) were significantly higher than those in the control group (704.81±291.72 μg/L) (p<0.001). No correlation was observed between soluble tumor necrosis factor-like weak inducer of apoptosis levels and ischemic area volume measured at diffusion-weighted magnetic resonance imaging (r=-0.008; p=0.07). The mean ischemic area volume was 505.68±381.10 and 60.96±80.89 mm^3^ in the nonsurviving and surviving patients, respectively (p=0.002).

**Conclusion:**

Soluble tumor necrosis factor-like weak inducer of apoptosis can be used in the diagnosis of acute ischemic stroke. However, it is inconclusive in estimating ischemic area volume and early mortality following acute ischemic stroke. Ischemic area volume measured at diffusion-weighted magnetic resonance imaging is a marker of poor prognosis and can be used in predicting early mortality.

Stroke with its two types (ischemic or hemorrhagic) is the second leading cause of mortality and the principal cause of long-term disability worldwide (1). The majority of strokes are acute ischemic stroke (AIS). Stroke is generally presented with a sudden onset of acute neurologic symptoms. Early diagnosis at admission and prompt restoration of normal blood flow are the key elements that determine the course of stroke patients (2). Cranial and neurovascular imaging is mandatory to confirm the preliminary diagnosis and selection of appropriate treatment strategies. The first step of current imaging practice for stroke is noncontrast computed tomography (CT) of the brain (3). It can be beneficial in detecting ischemic stroke changes besides its almost excellent accuracy for hemorrhagic stroke. Unfortunately, it is not very sensitive for minor ischemic strokes. By contrast, diffusion-weighted magnetic resonance imaging (DWI) can detect ischemic changes in the brain within minutes and show even small infarct areas (4,5). Therefore, it is more precise than CT for detecting the exact boundaries of an ischemic area volume (IAV) and more useful during follow-up.

Soluble tumor necrosis factor-like weak inducer of apoptosis (sTWEAK) is a growth regulator protein with weak apoptotic activity and a member of the tumor necrosis factor-α family. sTWEAK is involved in the regulation of several biological functions, including inflammatory cytokine release, cell growth, and angiogenesis induction, and particularly the stimulation of apoptosis (6). Fibroblast growth factor-inducible 14 (Fn14) is a type 1 transmembrane protein with a physiological affinity for sTWEAK and is a sTWEAK receptor (7). Studies have shown that sTWEAK and Fn14 are found in the heart, kidneys, endothelial and blood cells, and astrocytes, microglial cells, and neurons of the central nervous system (8,9,10,11). Inta et al. (11) showed that patients with AIS had high sTWEAK levels. However, no correlation was found between sTWEAK and IAV during acute stroke in a recent study (12).

The present study aimed to determine sTWEAK levels in patients with AIS, evaluate the relationship with IAV determined at DWI, and investigate the early predictive value of sTWEAK for the prognosis of AIS.

## MATERIALS AND METHODS

### Study design

This prospective case-control study was conducted after the local institutional review board approval (no: 2015-20/07). Patients with AIS with onset of stroke symptoms ≤12 h and presenting to the emergency department (ED) of the institution between March 1, 2016 and January 1, 2017, were included. Written informed consent forms were obtained from the patients.

The demographic data of the patients (age, sex, history, presence of chronic disease, and medications used), admission Glasgow coma scale (GCS), admission modified Rankin scale (mRS) scores, laboratory results, sTWEAK levels, admission IAV at DWI, and 6-month mRS were recorded. Only age, sex, and sTWEAK levels were recorded in the sex- and age-matched control group.

Subjects with a history of stroke, disease that may affect sTWEAK levels (acute myocardial infarction, kidney, liver, or heart failure), age <18 years, or refusal to participate, were excluded. The same exclusion criteria were also used for the control group.

The mortality and morbidity outcomes were classified as asymptomatic (mRS score of 0), mild sequela (mRS score of 1-2), moderate/severe sequela (mRS score of 3-5), and death (mRS score of 6) at 6-month mRS (180 days) following hospital discharge. The patients were followed-up prospectively by face-to-face or telephone interviews.

### Biochemical analysis

First, 10 mL blood specimens were collected and placed in biochemistry tubes. These were centrifuged for 6 min at 5000 rpm for serum separation. The sera obtained were then placed into Eppendorf tubes and stored at -80°C. The specimens were subsequently thawed simultaneously at room temperature when required for biochemical investigation. sTWEAK measurement was subsequently performed from serum specimens using the human TNFSF12/sTWEAK enzyme-linked immunosorbent assay (ELISA) kit (Boster Biological Technology, Pleasanton CA, USA) in line with the manufacturer’s instructions. The results were expressed as pg/mL. The human TNFSF12/sTWEAK ELISA kit measurement has a range of 62.5-10000 pg/mL, sensitivity of <10 pg/mL, intra-assay CV value of 5.4%, and inter-assay CV value of 6.4%.

### Diffusion-weighted magnetic resonance imaging

DWI was performed to patients presenting to the ED due to stroke within the first 12 h of arrival using a 1.5-T Philips Achieva (Philips Medical Systems, Best, Netherlands) device (with b values of 0 and 1000 s/mm^2^). Sequence duration was set at 43 s. DWI sequence parameters were TR/TE, 7216/122.8; flip angle, 90°; FOV, 24×24 cm; and matrix dimension, 128×128 mm. Section thickness during imaging was 5 mm, the intersection interval was 1 mm, and an average of 20-24 axial sections was obtained from each participant. DWI images were evaluated by the same two radiologists, and IAV was calculated according to the modified ellipsoid method by Sims et al. (13).

### Statistical analysis

SPSS for Windows 25.0 (SPSS Inc., Chicago, IL, USA) software was used for data analysis. Descriptive statistics was expressed as mean ± standard deviation. Power analysis was conducted for the independent groups by using t-test (Student t-test; i.e., the parametric alternative of Mann-Whitney U test) to determine the sample size before the study. Results indicated that the study should be conducted with 72 subjects, with medium effect size, 80% power, and a significance level of 0.05. Pearson chi-squared and Fisher Exact were used to analyze categorical variables. The Mann-Whitney U test was used to assess the significant difference between two independent groups in case of non-normally distributed data. Correlations between variables were evaluated using Spearman rho correlation analysis. sTWEAK sensitivity, specificity, positive and negative predictive values, and usefulness in the diagnosis of stroke and early prognosis were evaluated using receiver operating characteristic (ROC) curve analysis. An error probability of 5% (p<0.05) was considered statistically significant.

## RESULTS

During the study period, 112 stroke patients presented to the ED. Of these, 31, 7, 13, 20, and 5 were excluded because of presentation >12 h after onset of stroke symptoms, hemorrhagic stroke, history of stroke, comorbid diseases capable of affecting sTWEAK levels, and unwillingness to participate, respectively. Thus, this case-control study included 36 adult patients with AIS and 36 healthy volunteers.

The mean ages of the patient and control group were 66.06±14.59 years (range: 23-94 years) and 66.1±14.4 years (range: 25-94 years) (p=0.980), respectively, and 63.8% of both groups were men. Four in five patients (80.6%) presented to the ED within the first 3 h after onset of AIS symptoms, with most presenting with two neurological symptoms. The most common neurological examination findings were lateralizing neurological deficit, followed by lateralizing neurological deficit + speech disorder. Serum sTWEAK levels in the AIS group (1968.08±1441.99 μg/L) were significantly higher than those in the control group (704.81±291.72 μg/L) (p<0.001). The mean IAV in the AIS group was 159.78±263.11 mm^3^ (Table 1).

**Figure 1:**
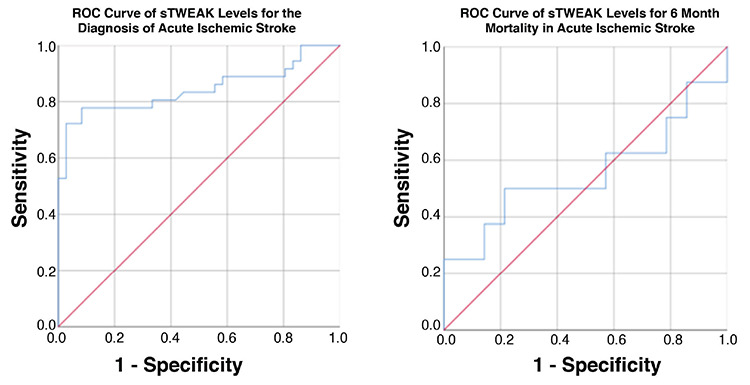
ROC curves of sTWEAK levels for the diagnosis of acute ischemic stroke and 6-month mortality in acute ischemic stroke.

At ROC curve analysis, sTWEAK emerged as a valuable marker in AIS prediction [area under curve (AUC): 0.84 (95% confidence interval, CI: 0.74-0.94; p<0.001)], with a cut-off value of 995.5 pg/mL, exhibiting 77.8% sensitivity, 91.7% specificity, 90.3% positive predictive value, and 80.5% negative predictive value. However, sTWEAK had no value in predicting 6-month mortality in patients with AIS [AUC: 0.55 (95% CI: 0.30-0.83; p=0.792)] (Figure 1).

No significant relationship was determined between sTWEAK levels and IAV (r=-0.008; p=0.07) or between sTWEAK levels and 6-month mRS (r=-0.04; p=0.65). GCS and mRS had a significant correlation with IAV (r=-0.66 and 0.63; p<0.01 and p<0.01, respectively) in patients with AIS. Admission GCS was also related with 6-month mRS (r=-0.81; p<0.01) (Table 2).

None of the patients underwent thrombectomy. Only nine patients in the AIS group were administered tissue plasminogen activator. Mortality occurred in 22.2% of patients with AIS during the first 6 months. GCS was significantly low, and IAV was significantly high in the nonsurviving group, whereas sTWEAK levels did not differ between the surviving and nonsurviving groups (p=0.648) (Table 3).

## DISCUSSION

A biomarker is any objectively evaluated parameter that reveals information about the diagnosis or course of a condition. The current diagnostic and prognostic biomarkers for AIS are based on imaging studies that might be unavailable for some patients or insufficient for minor cases. Imaging modalities also require physicians who are capable of evaluating them. The clinical practice of stroke is unfortunately short of objective, simple, easy to evaluate, and highly efficient biochemical biomarkers.

Several studies have been conducted to investigate the rapid diagnosis of AIS and the prediction of neurological deficit or mortality. Some of the markers whose value in diagnosis and predicting mortality and prognosis that have recently been investigated are glucose, iron, ferritin, homocysteine, insulin, P-selectin, matrix metalloproteinase-9, high-density lipoprotein cholesterol, platelets, C-reactive protein, glial fibrillary acidic protein, TNF-α, interleukin-6, and proenkephalin-A (14,15,16,17,18,19). Recently, sTWEAK has become a highly investigated protein, mainly due to its effect on apoptosis stimulation and inflammatory cytokine release (6,7,8,9,10,11). Literature shows that sTWEAK levels could be used as a marker of mortality and prognosis in patients with chronic kidney failure, non-ischemic heart failure, and chronic stable heart failure (20,21,22). Moreover, sTWEAK levels in patients with ST-elevation myocardial infarction (STEMI) is significantly higher compared with both healthy controls and subjects with stable coronary artery disease (23). In their study comparing sTWEAK levels of patients with abdominal aortic aneurysm (AAA) with those of healthy controls, Martin-Ventura et al. (24) reported an inverse correlation between low sTWEAK levels and aortic diameter. Therefore, they suggested that sTWEAK can be used to determine the diagnosis and prognosis of AAA (24). With regard to the potential of sTWEAK in central nervous system diseases, Inta et al. (11) examined the correlation between AIS and sTWEAK and determined that sTWEAK and Fn14 levels were significantly higher in patients with AIS presenting within 24 h compared with the control group. Although the high levels were correlated with post-stroke survival, no association was determined with IAV (11). In our study, sTWEAK levels in patients with AIS were significantly higher compared with healthy volunteers. Although this suggests that sTWEAK may have diagnostic value in AIS, we also determined that measuring sTWEAK levels was ineffective in terms of predicting mortality or prognosis.

The cut-off value is a crucial point for studies investigating serum biomarkers. Filusch et al. (25) reported a cut-off value of 306 pg/mL for sTWEAK in patients with pulmonary artery hypertension, whereas Chorianopoulos et al. (23) calculated the cut-off value as 1286 pg/mL in STEMI (25). We determined a cut-off value of 995.5 pg/mL for sTWEAK in patients with AIS. We attribute the differences in the cut-off values to the variety of the study groups and/or methodologies used. The IAV at DWI is currently used as a guide for both mortality and estimation of functional outcomes (26).

IAV is another parameter investigated in studies focusing on AIS. Morita et al. showed a correlation between neurological outcomes and IAV measured 3-24 h after AIS. In comparison, Thijs et al. reported a correlation between neurological outcomes with survival and IAV 48 h after AIS and concluded that IAV measured after stroke was an independent marker of post-stroke outcomes (27,28). In a study similar to ours, Yilmaz et al. (12) evaluated the relationship between sTWEAK, lesion size, and diagnostic value of sTWEAK in AIS cases. They concluded that sTWEAK could be a useful parameter for the diagnosis of acute stroke but did not correlate with IAV (12). In our study, 80.6% of patients presenting to the ED due to stroke were admitted within 3 h of onset of symptoms, and DWI was performed within 12 h after presentation. The IAVs of patients who died within 6 months, in particular, were significantly higher than those of the surviving patients. This finding is parallel with other studies reporting that IAV is a guide to prognosis, and it correlates with early-period survival.

Neurological scoring and laboratory results are two other variables, the effectiveness of which has been investigated in terms of survival and prognosis of stroke patients. However, there is no consensus regarding the efficacy of these variables. Lin et al. (29) reported that a GCS score of ≤12 was a marker of early neurological worsening. Yilmaz et al. (12) found that National Institutes of Health Stroke Scale score had a positive correlation with the IAV of stroke patients. In our study, GCS of ≤11 at the time of presentation to the ED was related with increased risk of 6-month mortality in patients with AIS. Hence, we suggest that low admission GCS following AIS is related to the patient’s mortality and prognosis.

As mentioned before, many system pathologies might result in changes in sTWEAK levels. Moreover, the etiology of AIS is highly related with kidney and liver diseases and cardiovascular problems (such as acute/chronic kidney disease, acute liver failure, arrhythmia, coronary artery disease, heart failure), each of which may lead to alteration in sTWEAK levels. In our study, the exclusion of the potential comorbidities associated with sTWEAK level alterations caused the decrease in the number of study patients. Another limitation of the present study might be the turnaround time of sTWEAK because the analysis of sTWEAK ELISA test generally takes approximately 2 h with the current techniques.

sTWEAK may function as a useful biomarker in the diagnosis of AIS. However, sTWEAK measurement is ineffective in predicting IAV or early-period mortality following AIS. Further studies are required for a better understanding of the relationship between the value of sTWEAK and AIS.

## Figures and Tables

**Table 1:**
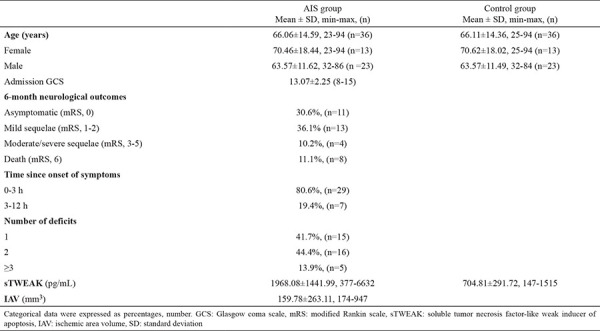
Demographic data, sTWEAK levels, and ischemic area volume results of the participants.

**Table 2:**
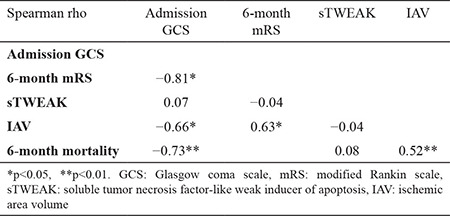
Bivariate correlations among GCS, 6-month mRS, sTWEAK, and ischemic area volume.

**Table 3:**
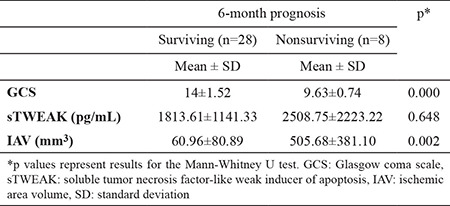
The 6-month mortality of patients with acute ischemic stroke.
